# Nasal Fish Tank Granuloma: An Uncommon Cause for Epistaxis

**DOI:** 10.4269/ajtmh.2011.11-0197

**Published:** 2011-08-01

**Authors:** Wan-Ling Ho, Wen-Yu Chuang, An-Jing Kuo, Kai-Chieh Chan

**Affiliations:** Department of Pediatrics, Shin Kong Wu Ho-Su Memorial Hospital, Taipei, Taiwan; Fu Jen School of Medicine, Catholic University, New Taipei City, Taiwan; Department of Pathology, Chang Gung Memorial Hospital and Chang Gung University, Linkou, Taiwan; Department of Medical Biotechnology and Laboratory Science, Chang Gung Memorial Hospital, Linkou, Taiwan; Department of Otolaryngology, Chang Gung Memorial Hospital and Chang Gung University, Linkou, Taiwan

**Figure 1.**
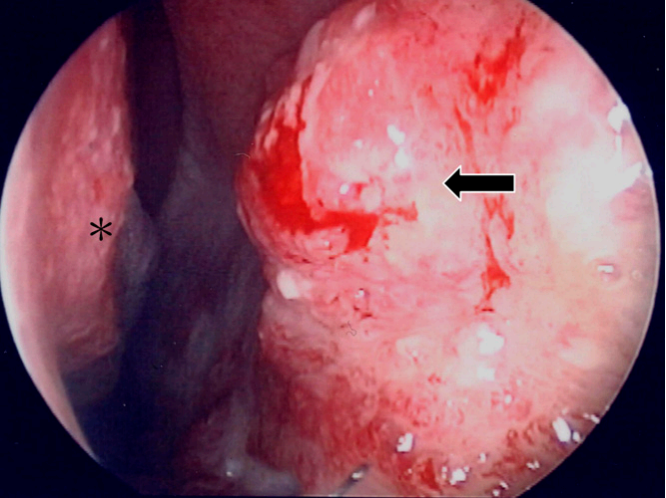
Anterior rhinoscopy revealed a granulomatous lesion with active oozing (arrow) on the right nasal septum. Inferior turbinate was identified as an asterisk.

**Figure 2.**
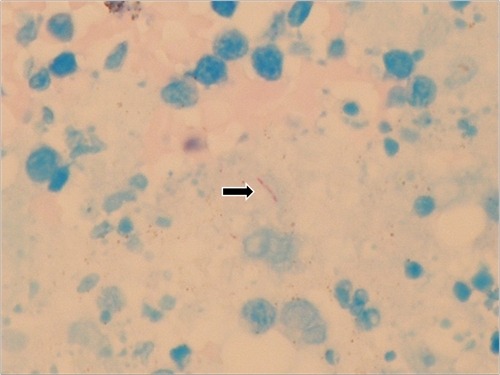
Histopathology of the specimen disclosed acid-fast bacilli (arrow) with the granulation inflammation.

**Figure 3.**
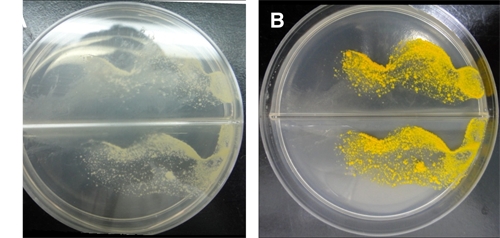
*Mycobacterium marinum* clones on Middlebrook 7H11 agar plates showed the photochromogenic characteristic, which are white when grown in the dark (**A**) and turn a brilliant yellow soon after exposure to light (**B**).

An immunocompetent 28-year-old male aquarium store employee presented with 1 month of intermittent epistaxis. A bleeding granulomatous lesion was identified over the right nasal septum (Figure 1). Histopathology from the incisional biopsy revealed granulomatous inflammation with the presence of acid-fast bacilli (Figure 2). Culture of the homogenized specimen on Middlebrook 7H11 agar after 14 days displayed white clones of non-tuberculous *Mycobacterium* (NTM) that turned brilliant yellow upon light exposure (Figure 3). Polymerase chain reaction (PCR) amplification of the 65-kDa heat shock protein gene with restriction enzyme analysis using *Bst*E II and *Hae* III confirmed the diagnosis of *Mycobacterium marinum* infection. The lesion regressed completely with 3 months of doxycycline treatment.

Fish tank granuloma is a rare granulomatous skin infection caused by *Mycobacterium marinum*, an opportunistic NTM, commonly found in aquatic environments.^1^ Extremities are most frequently affected; nasal cavity infection is seldom reported. The diagnosis is generally based on a history of aquatic exposure (occupational or recreational), histopathology of granulomatous inflammation with or without the presence of acid-fast bacilli, and a slow-growing photochromogenic NTM incubated at low temperature (28–30°C). Furthermore, PCR facilitates an early identification of *Mycobacterium marinum* infection. Superficial lesion sometimes resolves spontaneously, but several months of antimicrobial drug are usually necessary. Various monotherapies, including doxycycline, are effective.^2^
